# Quantitative Analysis of the Time–Intensity Curve of Contrast-Enhanced Ultrasound of the Liver: Differentiation of Benign and Malignant Liver Lesions

**DOI:** 10.3390/diagnostics11071244

**Published:** 2021-07-12

**Authors:** Sonja Schwarz, Dirk-André Clevert, Michael Ingrisch, Thomas Geyer, Vincent Schwarze, Johannes Rübenthaler, Marco Armbruster

**Affiliations:** Department of Radiology, University Hospital, LMU Munich, Marchioninistr. 15, 81377 München, Germany; sonjaschwarz128@gmail.com (S.S.);Dirk.Clevert@med.uni-muenchen.de (D.-A.C.); Michael.Ingrisch@med.uni-muenchen.de (M.I.); Thomas.Geyer@med.uni-muenchen.de (T.G.); Vincent.Schwarze@med.uni-muenchen.de (V.S.); Johannes.Ruebenthaler@med.uni-muenchen.de (J.R.)

**Keywords:** liver diagnostic imaging, neoplasm, ultrasonography, image enhancement, image processing, computer-assisted

## Abstract

Background: To evaluate the diagnostic accuracy of quantitative perfusion parameters in contrast-enhanced ultrasound to differentiate malignant from benign liver lesions. Methods: In this retrospective study 134 patients with a total of 139 focal liver lesions were included who underwent contrast enhanced ultrasound (CEUS) between 2008 and 2018. All examinations were performed by a single radiologist with more than 15 years of experience using a second-generation blood pool contrast agent. The standard of reference was histopathology (*n* = 60), MRI or CT (*n* = 75) or long-term CEUS follow up (*n* = 4). For post processing regions of interests were drawn both inside of target lesions and the liver background. Time–intensity curves were fitted to the CEUS DICOM dataset and the rise time (RT) of contrast enhancement until peak enhancement, and a late-phase ratio (LPR) of signal intensities within the lesion and the background tissue, were calculated and compared between malignant and benign liver lesion using Student’s *t*-test. Quantitative parameters were evaluated with respect to their diagnostic accuracy using receiver operator characteristic curves. Both features were then combined in a logistic regression model and the cumulated accuracy was assessed. Results: RT of benign lesions (14.8 ± 13.8 s, *p* = 0.005), and in a subgroup analysis, particular hemangiomas (23.4 ± 16.2 s, *p* < 0.001) differed significantly to malignant lesions (9.3 ± 3.8 s). The LPR was significantly different between benign (1.59 ± 1.59, *p* < 0.001) and malignant lesions (0.38 ± 0.23). Logistic regression analysis with RT and LPR combined showed a high diagnostic accuracy of quantitative CEUS parameters with areas under the curve of 0.923 (benign vs. malignant) and 0.929 (hemangioma vs. malignant. Conclusions: Quantified CEUS parameters are helpful to differentiate malignant from benign liver lesions, in particular in case of atypical hemangiomas.

## 1. Introduction

Focal liver lesions (FLL) are common and can be found in about 5% of the European population [1]. FLL are frequently detected incidentally by ultrasound examination of the upper abdomen or during computed tomography (CT) and magnetic resonance imaging (MRI) scans. It is very important to differentiate benign from malignant liver lesions quickly and safely to ensure a correct therapeutic management and to avoid unnecessary invasive procedures and psychological stress for the patient. Due to its high availability, B-mode sonography is considered the first diagnostic step in the workup of FLL as it is uncomplicated, cost-effective and radiation-free. It is also used to screen for malignant FLL like hepatocellular carcinoma (HCC) following the guidelines of the European Association for the Study of the Liver (EASL) [2].

Contrast-enhanced ultrasound (CEUS) can be performed within the same investigation as B-mode and Doppler ultrasound and provides additional information about the vascularization and contrast dynamics of FLL with a good diagnostic performance comparable to CT and MRI [3,4,5]. A focal liver lesion found in conventional ultrasound can therefore in many cases be diagnosed immediately via CEUS without another appointment at CT or MRI, sparing patients psychical stress and, in case of CT, radiation exposure. The latter especially affects young patients with frequent follow ups. Furthermore, CEUS is also suitable in patients who have contraindications to MRI like pacemakers or defibrillators. The contrast agent used for sonography is very well tolerated and unlike in CT or MRI there are no contraindications regarding hyperthyroidism or renal insufficiency. Additionally, CEUS provides the unique feature to visualize contrast dynamics in real-time and therefore gives a dynamic impression of the timing and intensity of washout [6,7]. A detailed literature review by Westwood et al. reported lower costs with a similar diagnostic performance of CEUS compared to contrast enhanced CT and MRI in the surveillance of cirrhosis and colorectal carcinoma [8].

To distinguish benign from malignant FLL in CEUS, specific contrast enhancement patterns are used [9]. The Guideline update 2012 of the World Federation for Ultrasound in Medicine and Biology (WFUMB) and the European Federation of Societies for Ultrasound in Medicine and Biology (EFSUMB) state that hypoenhancement of solid lesions in the late and postvascular phases characterizes malignancies, and that almost all metastases and typical HCCs show this feature [10]. However, the distinction of benign lesions with mild hypoenhancement in the late phase, or lack of enhancement because of thrombosed portions, can be particularly challenging [10]. In such cases quantitative analysis of time–intensity curves in CEUS could provide further information and might overcome the limitations of a purely visual assessment. Furthermore, quantification enables computer-aided diagnoses in the long term and objectifies the very examiner-dependent CEUS examination and interpretation. In a former study Wilson SR et al. reported the value of qualitative assessment of CEUS using an algorithm-based approach to differentiate malignant from benign liver lesions and achieved a good diagnostic accuracy of 85–92% [11,12]. Single studies with computer-aided evaluations, such as the classification algorithm of Gatos et al. (*n* = 52), reported a diagnostic accuracy up to 90.3% [13]. On the other hand, existing clinical trials analyzing CEUS perfusion quantification parameters showed varying results with only few providing meaningful case numbers. While Beyer et al. distinguished benign and malignant liver lesions by quantifying regional blood flow, regional blood volume, and peak enhancement with receiver operator characteristic curves (ROC) of 0.97, 0.96, 0.98, and 0.76, respectively (*n* = 20) [14], Goertz et al. found no significant difference between benign FLL and malignancies in peak enhancement [15].

In order to evaluate CEUS perfusion quantification parameters in a larger cohort and to evaluate which parameters are best suited to differentiate malignant from benign liver lesions we retrospectively evaluated 139 CEUS examinations with histopathology, MRI or long-term follow-up as the standard of reference.

## 2. Materials and Methods

### 2.1. Study Cohort

In this retrospective study, 139 FLL from 134 patients who underwent CEUS between March 2008 and September 2018 at our institution were analyzed by quantifying contrast enhancement in the arterial and late phase. Written consent was obtained from all patients prior to CEUS. Seventy-five FLLs were found in women (mean age: 55 ±18 years; range: 24–93 years) and 64 in men (mean age: 64 ± 15 years; range: 21–99 years).

**Figure 1:**
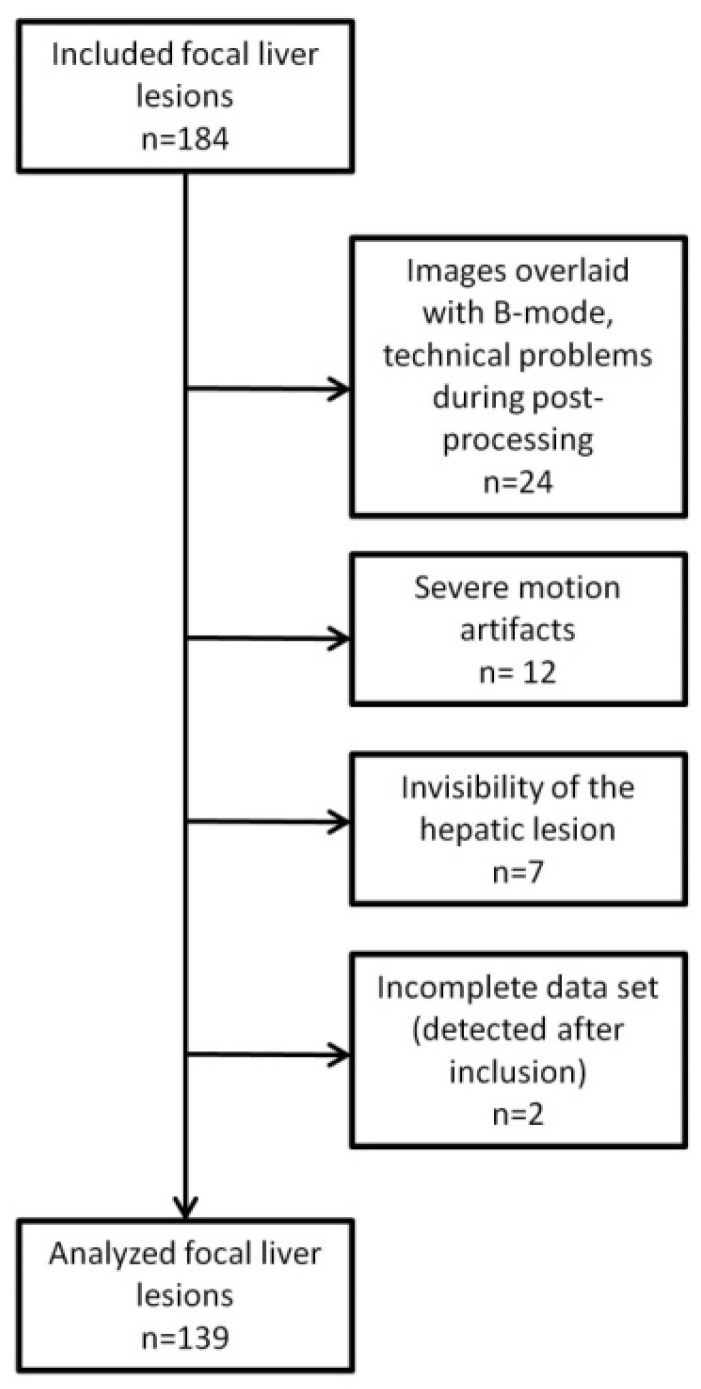
Flow chart of the study population and excluded cases.

Inclusion criteria were known hepatic lesion other than simple liver cyst, histopathology, or contrast-enhanced CT, MRI or PET/CT serving as standard of reference, and available CEUS DICOM clips in our picture archive. Regarding CEUS dataset either the arterial phase had to be long enough to include both the arrival of the contrast medium as well as the peak enhancement, or an additional clip during the portal venous phase had to exist, furthermore at least one clip during the late phase was mandatory. Cases were excluded, when CEUS datasets were only available in overlay to B-mode images due to technical problems during post-processing (*n* = 24), due to severe motion artifacts (*n* = 12), invisibility of the hepatic lesion in CEUS (*n* = 7) or due to incomplete CEUS dataset (*n* = 2) (Figure 1).

### 2.2. CEUS

Contrast-enhanced ultrasound was performed by one skilled radiologist with experience since 2000 (EFSUMB Level 3). All CEUS examinations were performed with up-to-date ultrasound devises (ACUSON Sequoia, S2000 or S3000—Siemens Healthineers, Mountain View, CA, USA; EPIQ 7—Philips, Seattle, WA, USA). Siemens systems provided C4-1 and C6-1 HD transducers and the Philips system provided a C9-2 transducer. All examinations were carried out with low mechanical index (<0.2) to prevent early destroying of the microbubbles.

After B-mode ultrasound and Color Doppler, 2.4 mL of Sulphur hexafluoride microbubbles (SonoVue, Bracco International B.V., Milan, Italy) were injected to a cubital vein followed by a flush of 5–10 mL saline 0.9%. Data sets of the arterial phase were recorded from the first arrival of contrast agent in the liver vessels. To reduce the destruction of the microbubbles, transducers were switched on only temporally for about 30 s to evaluate contrast enhancement during each the arterial (10–45 s), portal (30–120 s) and late phases (120–180 s). No adverse effects were registered during and after CEUS examinations.

### 2.3. Quantitative CEUS Assessment

**Figure 2:**
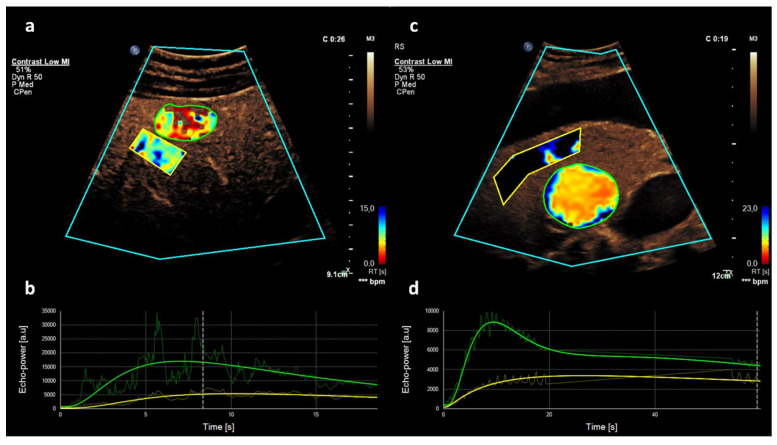
Rise time (RT) in hemangioma and hepatocellular carcinoma. CEUS of the liver with color maps, from red (fast enhancement) to blue (slow enhancement) (**a**,**c**) and signal intensity curves (**b**,**d**) during the arterial phase in a 59-year-old male patient with liver hemangioma and steatosis hepatis (**a**) in comparison to a 67-year-old male patient suffering from hepatocellular carcinoma and liver cirrhosis (**c**). The RT of contrast enhancement until the peak enhancement (green curves) differs significantly between the two different target lesions (green) and the surrounding normal liver tissue (yellow). While in hemangioma the curve rises and flattens slowly (**b**), in HCC the RT is much shorter.

In order to process and analyze the datasets, we used available proprietary software (VueBox; Bracco, Suisse SA, Plan-les-Ouates, Genève-Switzerland). For each of the transducers listed above, a calibration file was applied, afterwards delimitation region of interest (ROI) was set for each uncompressed DICOM cine loop and motion correction was performed using a retrospective trigger box over the lung and liver. A ROI was then manually drawn within the target lesion. The entire lesion was included in the ROI, sparing necrotic parts, large vessels or thrombosis that otherwise likely would confound the analysis. In addition, we selected a reference region (REF) within the surrounding, normal-appearing liver tissue in the same depth from the transducer as the target lesion (Figure 2).

Both within the target lesion and REF, the brightness of the pixels was fitted over time resulting in time–intensity curves of perfusion. Both the rise time (RT) of contrast enhancement and a late phase ratio (LPR) were calculated. The RT describes the early contrast dynamics until peak enhancement. Its starting point is calculated from the enhancement curve as intersection of a tangent to the maximum slope with the time axis [16]. The LPR quantifies the contrast enhancement in the late phase as hypo-, iso- or hyperenhancement calculating the signal intensity ratio ROI/REF during the late phase.

The statistical analysis was performed with MS Excel 2007 (Microsoft Corporation, Redmond, WA, USA) and Stata-IC15.1 (StataCorp LLC, College Station, TX, USA). For descriptive statistics means ± standard deviations (SD) of all quantitative perfusion parameters were calculated. Benign and malignant FLL, as well as hemangiomas in particular and malignant FLL, were studied and compared. To test for differences of perfusion parameters Student’s *t*-test and ROC analyses were applied. Tests were considered significant at *p* < 0.05. For each parameter a cut-off was defined. Sensitivity, specificity, accuracy, positive likelihood ratio (LR+) and negative likelihood ratio (LR−) were calculated.

To evaluate the relationship between the likelihood of a lesion being benign, or an hemangioma, and the US-derived measures RT and LPR, a logistic regression model

$$p\left(y=1\right)=\frac{e^{ß0+ß1\times LPR+ß2\times RT}}{1+e^{ß0+ß1\times LPR+ß2\times RT}}$$

was fitted to the observed data. The statistical analysis was performed with the logistic function in Stata-IC15.1 (StataCorp LLC, College Station, TX, USA) [17]. To illustrate the predictive potential, another ROC analysis was performed directly on the logistic regression results. The AUC was again used as a measure of diagnostic performance.

## 3. Results

### 3.1. Patient Characteristics

**Figure 3:**
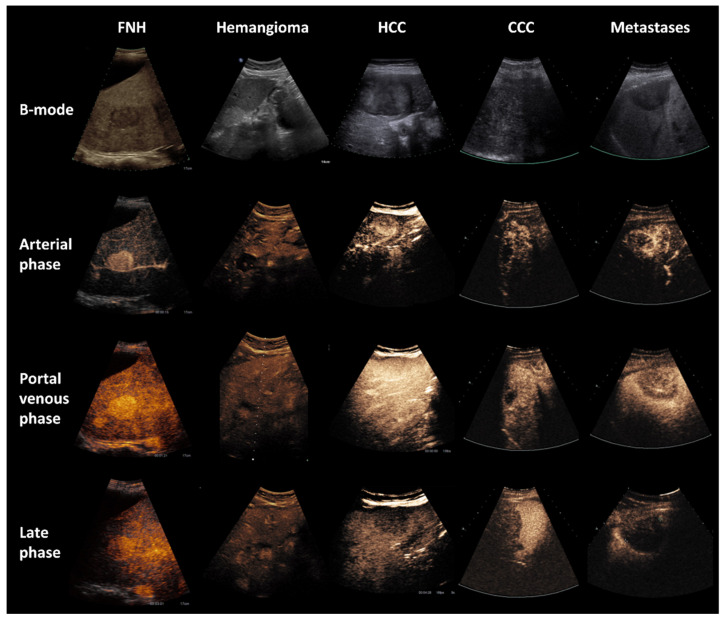
B-mode and CEUS of the liver in different focal liver lesions. 1st column: CEUS in a 26-year-old female with focal nodular hyperplasia (FNH); Bright hyperenhancement of contrast agent in all phases. 2nd column: CEUS in a 39-year-old male with hemangioma; Slow contrast enhancement in the arterial and portalvenous phase, approximate isoenhancement in the late phase. 3rd column: CEUS in a 51-year-old female with hepatocellular carcinoma (HCC); Bright contrast enhancement in arterial and portalvenous phase, wash-out in the late phase. 4th column: CEUS in a 42-year-old female with cholangiocellular carcinoma (CCC); Contrast enhancement in arterial and portalvenous phase, wash-out in the late phase. 5th column: CEUS in a 54-year-old female with liver metastases of breast cancer; Contrast enhancement in the arterial phase, wash-out with beginning in the portalvenous phase.

Following our inclusion and exclusion criteria, we included 139 FLL in 134 patients (female/male = 75/64). Forty-four benign liver lesions were examined including focal nodular hyperplasia (FNH; *n* = 20), hemangioma (*n* = 16) and adenoma (*n* = 8). The group of the malignant FLL (*n* = 95) comprised hepatocellular carcinoma (HCC; *n* = 30), cholangiocellular carcinoma (CCC; *n* = 16) and metastases (*n* = 49) (see example images in Figure 3). The standard of reference for the classification of FLL was histopathology (*n* = 60), imaging other than CEUS (*n* = 75) or follow up (*n* = 4). For precise distribution of age, tumor entity, and reference standard see Table 1. The arterial enhancement phase was feasible for scrutiny in 90 cases, the late phase in 134 cases (see Table 1). In 85 cases both arterial and late phase were analyzed.

### 3.2. Late Phase Ratio

**Figure 4:**
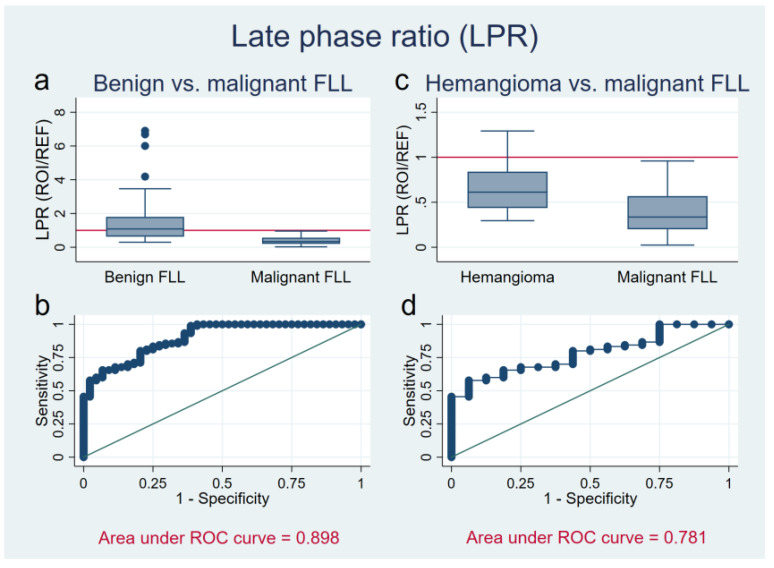
Late phase ratio (LPR) of benign liver lesions (**a**,**b**), and in a subgroup analysis of hemangiomas (**c**,**d**) versus malignant focal liver lesions (FLL). (**a**,**c**) The *Y* axis shows the LPR (region of interest/reference) of benign (**a**) FLL and hemangiomas (**c**) in comparison to malignant FLL. Red reference line at 1, values below indicating hypoechogenicity, values above hyperechogenicity. For malignant tumors, the ratio lies below 1 and therefore differ significantly (*p* < 0.001) from benign FLL (**a**). Hemangiomas present variable, in many cases mildly hypoechoic. Still, they differ significantly from malignancies (*p* < 0.001) (**c**). (**b**,**d**) ROC curve of LPR (*X* axis: 1-Specifity; *Y* axis: Sensitivity) in discriminating malignant from benign liver lesions (Area under ROC curve = 0.898) (**b**), and malignant lesions from hemangiomas (Area under ROC curve = 0.781) (**d**).

Signal intensity in the late phase was assessed in relation to the surrounding parenchyma by the LPR (ROI/REF). LPR was 0.38 ± 0.23 (range: 0.02–0.96) for malignant lesions with a ratio less than one for all FLL apart from one single value in the CCC group, which was isoechogenic (0.96) (see Figure 4). For benign FLL hypo-, iso- and hyperenhancement occurred with a LPR of 1.59 ± 1.59 (range: 0.30–6.90). As expected FNHs showed a strong contrast enhancement in the late phase (LPR: 2.42 ± 2.00, range: 0.43–6.90) in the vast majority of cases while adenomas were almost always isoechogenic, sometimes with mild hypo- or hyperenhancement (LPR: 1.34 ± 0.77, range: 0.54–2.47). Hemangiomas differed in their appearance and were mildly hypoechogenic in the late phase with LPR: 0.67 ± 0.30 (range: 0.30–1.29).

Altogether, the LPR of benign entities significantly differed from malignant entities in the late phase (*p* < 0.001). Calculating the ROC curve of LPR resulted in an area under the curve (AUC) of 0.898 (see Figure 4). With a cut off at 1.0 (ROI/REF), every measurable hypoechogenicity is suspicious for malignancy. The sensitivity in this case is at 100% with a specificity of 56.8%, accuracy of 85.8%, LR+ of 2.3 and LR- of 0.0.

Focusing on hemangiomas, those lesions which showed only slight hypoechoic appearance in comparison to the surrounding tissue in the late phase (LPR: 0.67 ± 0.30; range: 0.30–1.29) differed significantly from the hypoechogenicity of malignant lesions which were markedly hypoechoic (LPR: 0.38 ± 0.23; range: 0.02–0.96; *p* < 0.001) and must not be confused with a real washout of contrast media. This pseudo-washout appearance is thought to be due to microbubble rupture within the hemangioma in case of a prolonged insonation. Similar findings were reported from Gianetti et al. [18]. The ROC curve in discriminating hemangioma from malignant FLL resulted in an AUC of 0.781(see Figure 4). Assuming a LPR of 0.6 as cut off, i.e., strong hypoechogenicity, results in a sensitivity for malignancy of 83.3%, a specificity of 37.5%, diagnostic accuracy of 76.4%, LR+ of 1.3 and LR- of 0.4.

### 3.3. Rise Time

**Figure 5:**
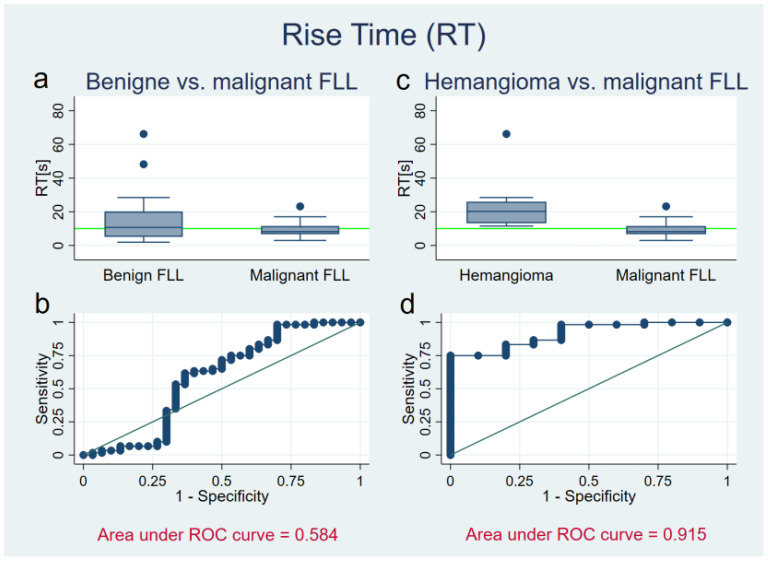
Rise time (RT) of benign liver lesions (**a**,**b**), and in a subgroup analysis of hemangiomas (**c**,**d**) versus malignant focal liver lesions (FLL). (**a**,**c**) The *Y* axis shows the RT of benign (**a**) FLL and hemangiomas (**c**) in comparison to malignant FLL. The green reference line is at 10 s. All hemangiomas lie over it, most malignancies lie beneath. Benign lesions present a wide spreading. (**b**,**d**) ROC curve of RT (*X* axis: 1-Specifity; *Y* axis: Sensitivity) in discriminating malignant from benign liver lesions (Area under ROC curve = 0.584) (**b**) and malignant lesions from hemangiomas (Area under ROC curve = 0.915) (**d**).

Rise time for benign FLL (hemangioma, FNH, and adenoma) was 14.8 ± 13.8 s (range: 1.9–66.2 s). Rise time for malignant FLLs was 9.3 ± 3.8 s (range: 3.0–23.2 s). In hemangiomas and liver cell adenomas it took significantly longer until the maximum contrast agent concentration was reached compared with other FLLs (adenoma: 22.8 ± 15.3 s, *p* = 0.002; hemangioma: 23.4 ± 16.2 s, *p* < 0.001), whereas FNHs (6.4 ± 0.7 s; *p* = 0.022) showed a faster enhancement than other FLLs (12.1 ± 9.4 s). The RT of hemangiomas was significantly longer than in malignant FLLs (*p* < 0.001) resulting in an AUC of 0.915. Values of RT less than 18.2 s as cut-off resulted in a sensitivity of 98.3%, specificity of 50.0%, diagnostic accuracy of 91.4%, a LR+ 2.0 and LR- of 0.0. When RTs of all benign lesions were compared to those of malignant tumors, values were also significantly different (*p* = 0.005), however ROC analysis revealed only a weak diagnostic performance resulting in an AUC of 0.584 (see Figure 5). Therefore, it was not applicable to define a cut-off here.

### 3.4. Logistic Regression

**Figure 6:**
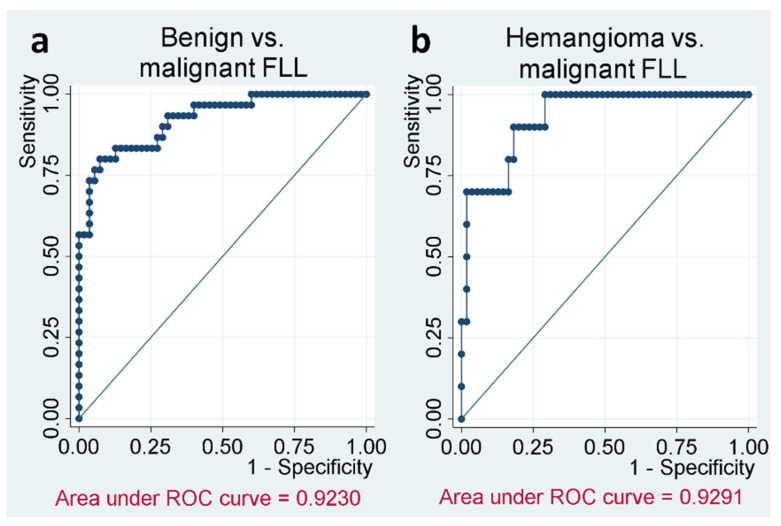
Rise time and late phase ratio combined in logistic regression (*X* axis: 1-Specifity; *Y* axis: Sensitivity). (**a**) The combination of parameters differs well between benign lesions and malignancies. The area under ROC curve is 0.923. (**b**) The combination of parameters differs well between hemangiomas and malignancies. The area under ROC curve is 0.929.

**Figure 7:**
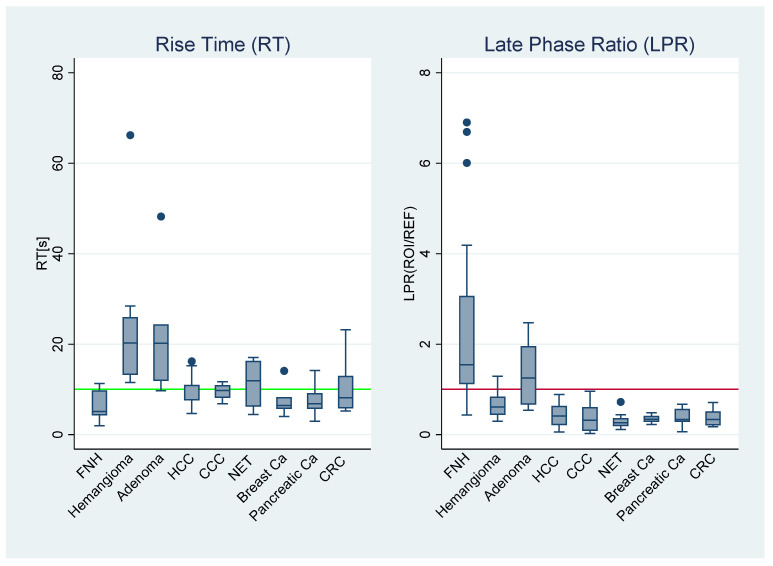
Box plots over all sub-entities. Rise time (RT) and late phase ratio (LPR) of all sub-entities. The green reference line is at 10 s, the red line at “1” distinguishes hyper- and hypoechogenicity of the liver lesions in the late phase. NET/Breast Ca/Pancreatic Ca/CRC denote the corresponding liver metastasis. FNH: focal nodular hyperplasia; HCC: hepatocellular carcinoma; CCC: cholangiocellular carcinoma; NET: neuroendocrine tumor; CRC: colorectal carcinoma.

Logistic regression analysis resulted in higher diagnostic performance for distinguishing benign and malignant FLL than each parameter individually with an AUC of 0.923. For differentiating hemangioma and malignant tumors, the combined area under the ROC curve was comparably good at 0.929 (see Figure 6). The log of the odds of a lesion being benign vs. malignant FLL were positively related to LPR (exp(ß1_LPR) = 380, 95% C.I. 20.8–6960, *p* < 0.001) and positively related to RT (exp(ß2_RT) = 1.2, 95% C.I. 1.02–1.30, *p* = 0.025). Likewise, for hemangioma vs. malignant FLL, LPR showed positive, but not significant, relation (exp(ß1_LPR) = 19.2, 95% C.I. 0.35–1060, *p* = 0.149) and RT was positively related to hemangioma (exp(ß2_RT) = 1.3, 95% C.I. 1.09–1.63, *p* = 0.004). An overview over the descriptive statistics of all different sub-entities is presented by the box plots of Figure 7.

## 4. Discussion

In this study, we retrospectively analyzed the quantified contrast enhancement pattern of different focal liver lesions (FLL) in CEUS, considering rise time (RT) and late phase ratio (LPR). Both RT and LPR significantly differed between benign and malignant tumors. The combination of both parameters in a logistic regression further improved the discrimination between those groups. The combination of RT and LRP was particularly useful to distinguish hemangiomas from malignancies.

LPR is a quantified parameter of contrast intensity obtained during the late phase, which is, in case of hypoenhancement, a well-established qualitative criterion for malignancy [10]. A significantly lower LPR was observed in malignant compared to benign focal liver lesions (FLL), congruent to the study of Goertz et al. 2010 (*n* = 33) [15]. Wildner et al. 2019 quantified late phase hypoenhancement in various tumor subgroups like focal nodular hyperplasia (FNH), hepatocellular carcinoma (HCC), cholangiocellular carcinoma, and metastases from cancer of the breast, pancreas, colon and melanoma (*n* = 148) [19] and found significant differences between FHN (higher signal intensity) and metastases of pancreatic and colorectal carcinoma. In most studies, only few benign lesions with hypoenhancement in the late phase were observed. Especially hemangiomas can be misinterpreted as malignant tumors because of their slight late phase hypoenhancement [10,20,21]. In our study, several hemangiomas with an LPR smaller than one were found, which, however, must not be interpreted as a classical wash-out phenomenon, since those hemangiomas are often only slightly vascularized and already appear isoechogenic during the arterial phase. Even if they didn’t lose contrast over the time, they might appear darker than the surrounding tissue, simulating wash-out which could confuse for malignancy. Our results show that there is still a measurable, significant difference between hemangiomas and malignant FLL using the LPR, which is smaller in malignancies and a cut off at 0.6 was suggested to differentiate marked hypoechogenicity from pseudo wash-out.

The RT showed large variations within the heterogeneous group of benign lesions. In our study, RT was particularly long in hemangiomas and adenomas and shortest in FNHs. These findings are in accordance with former studies from Pei et al. 2013 [22], who found a significant shorter time to peak in FNH compared with HCC (*n* = 100), and Zheng et al. 2013 [23], who observed the same tendency (*n* = 60).

In general, it is assumed that a short RT is related to arterial hypervascularity [24], as the number of feeding arteries show a larger contribution to contrast inflow than sinusoidal capillarization [25]. In hemangiomas, the long RT reflects the well-known progressive centripetal fill-in during the extended portal–venous phase [21] and differs strongly from malignancies. In our study population RT was significantly longer for hemangioma (23.4 ± 16.2 s, *p* < 0.001) than for malignant lesions (9.3 ± 3.8 s) and could discriminate these two entities with a high diagnostic accuracy (AUC: 0.915). When comparing all benign to malignant liver lesions, RT showed only a moderate diagnostic performance (AUC: 0.584), which can be mainly explained by the short RT of FNH (6.4 ± 0.7 s) which appear different than most of the other benign liver lesions and therefore confound the quantitative assessment. To overcome these limitations, we suggest the following algorithm: if the LPR is >1 and the lesion visually presents hyperechoic, we mostly expect a benign focus. In this case a short RT speaks for FNH and strengthens the diagnosis. If the LPR is markedly <1, a diagnosis of malignancy is recommended. An LPR just little below 1 with only mild visual hypoechogenicity and a long RT strongly suggests hemangioma is the correct diagnosis. The good diagnostic performance of the combined parameters in this study is shown by the receiver operator curve (ROC) after logistic regression with an AUC for hemangioma vs. malignant FLL of 0.929 and for benign vs. malignant FLL of 0.923. However, further prospective studies are necessary to investigate the clinical value and diagnostic performance of the proposed diagnostic algorithm.

Its direct accessibility and repeatability, cost-effectiveness, and non-ionizing excellent safety profile make multiparametric CEUS a powerful imaging tool for assessing focal liver lesions [26]. Furthermore, safe application of CEUS in children and during pregnancy was recently described [27,28,29] and strengthens its pivotal role in assessing FLL also with respect to inclusion of CEUS in hepatic imaging guidelines in the future [2,30].

There are some limitations to this study. The lesions found in CEUS were evaluated according to clinical standards, e.g., histological diagnostic or further cross-sectional imaging. Especially typical benign tumors or HCC with typical imaging appearance in patients with cirrhosis do not justify an invasive proceed to confirm the diagnosis. Therefore, in four cases (three FNH, one adenoma), the diagnosis was confirmed as benign by constant CEUS follow-up with a stable size during a period of at least 24 months. In these cases, a repeated misdiagnosis within the benign spectrum is theoretically possible.

Our study population includes a great diversity in tumor diameter (0.6–10 cm), and pretreatments (see Table 1). Even if tumor size and previous treatments might influence the dynamic of the contrast enhancement [31,32], a systematic error is unlikely, as different tumor sizes were distributed almost evenly between benign and malignant lesions, while previous treatments (*n* = 15) were contributed comparably over the different malignant subgroups.

## 5. Conclusions

In conclusion, we were able to show that quantification of CEUS parameters with assessment of the rise time of contrast enhancement until its peak and calculation of a late phase lesion-to-background ratio is helpful to differentiate malignant from benign liver lesions, particularly in hemangiomas with slight hypoenhancement in the late phase and might contribute to an objective and more specific diagnosis of FLL in CEUS.

## Figures and Tables

**Table 1:** Study cohort (*n* = 139) with subgroups of different focal liver lesions.

Entity	*n*	Art. Phase	Late Phase	M/F	Age	Tumor Size	Cirrhosis	Evaluation			Previous Treatments
								Histo	CT/MR	Follow up	
**Total**	139	90	134	64/75	59 ± 17	3.4 ± 2.7	28	60	75	4	
FNH	20	15	20	3/17	39 ± 13	4.4 ± 2.6	0	3	14	3	
Hemangioma	16	10	16	5/11	50 ± 11	2.2 ± 1.9	1		16		
Adenoma	8	5	8	3/5	49 ± 23	2.6 ± 1.5	0	3	4	1	1× partial resection
HCC	30	17	28	24/6	62 ± 9	3.7 ± 2.8	26	9	21		2×TACE 1× liver transplant
CCC	16	13	14	8/8	68 ± 14	5.3 ± 3.2	0	16	0		2× partial resection, 1× PDT
**Metastasis**											
NET	14	7	14	8/6	62 ± 17	3.8 ± 3.7	0	8	6		3× RPT 1× hemi- hepatectomy1× TACE
Breast Ca	7	6	7	0/7	67 ± 14	2.8 ± 2.0	1	4	3		
Pancreatic Ca	14	9	13	6/8	69 ± 12	1.1 ± 0.3	0	9	5		1× liver transplant
CRC	14	8	14	7/7	70 ± 13	2.7 ± 2.0	0	8	6		2× partial resection

M/F: male/female; FNH: Focal nodular hyperplasia; HCC: Hepatocellular carcinoma; CCC: Cholangiocellular carcinoma; NET: neuroendocrine tumor; CRC: colorectal carcinoma; TACE: transarterial chemoembolization, PDT: photodynamic therapy, RPT: radiopeptide therapy.
